# The manufacturing of human viral challenge agents for use in clinical studies to accelerate the drug development process

**DOI:** 10.1186/s13104-018-3636-7

**Published:** 2018-08-29

**Authors:** Andrew P. Catchpole, Daniel J. Fullen, Nicolas Noulin, Alex Mann, Anthony S. Gilbert, Rob Lambkin-Williams

**Affiliations:** hVivo Services Ltd, London, UK

**Keywords:** Human viral challenge model, HVC, Experimental challenge, Viral challenge, Good manufacturing process, GMP, Adventitious agent testing, Clinical trials, Influenza, Human rhinovirus, Respiratory syncytial virus, Asthma

## Abstract

**Objective:**

This manuscript aims to provide an overview of the unique considerations and best practice principles associated with the manufacture of human viral challenge agents.

**Results:**

Considerations are discussed on the entire process from strain and viral source selection through manufacturing, safety and efficacy testing. The human viral challenge (HVC) model is an important tool to help accelerate the drug development process but producing viruses suitable for use in the model presents a unique set of challenges. There are many case by case decisions and risk assessments to consider and no clear international standard to produce viruses for this purpose. The authors present challenge virus manufacturing considerations from the current literature, regulatory guidance and their own direct experience in producing challenge viruses. The use of these viral stocks in clinical studies, as published in peer-reviewed journals, is also briefly described.

**Electronic supplementary material:**

The online version of this article (10.1186/s13104-018-3636-7) contains supplementary material, which is available to authorized users.

## Introduction

Edward Jenner performed possibly the first documented Human Viral Challenge (HVC) study in 1796 [1]; since then such studies have become established. A range of challenge agents (CAs) including viruses, toxins, allergens, bacteria and fungi have been administered to people to induce a physiological or pathological effect [2] under various regulatory frameworks. The US regulators consider CAs like drugs, and thus studies performed in the US are conducted within the FDA (US Food and Drug Administration) Investigational New Drug (IND) framework. The UK and the EU regulators don’t, resulting in less clarity regarding the guidance to follow [3, 4]. When a CA is used in the UK, not in conjunction with an investigational medicinal product (IMP), a study is classified as a clinical “study” and not as a clinical “trial” as defined in the Medicines Regulation 536/2014 [5].

Considering the lack of specific guidance on production of viral CAs, this publication aims to provide some guidance to others considering producing CAs by addressing the key points we considered in the production of our respiratory virus CAs. We incorporate reviews of the latest regulatory and GMP guidelines, the scientific literature and our own discussions with the FDA and MHRA. The authors have conducted multiple HVC studies with over 2500 volunteers using numerous different challenge viruses including influenza, respiratory syncytial virus (RSV) and human rhinovirus (HRV).

## Main text

Safety of challenge viruses is of paramount importance and when selecting a suitable virus, hence a thorough literature review of the naturally occurring pathogenicity of the virus and potential risks should be undertaken. As an example; (H1N1) A/California/7/2009-like (pdm09-like) influenza virus isolates may have two key mutations in the Haemagglutinin receptor binding site, D222G and D222N. Both lead to greater tropism for the lower respiratory tract and are associated with severe clinical outcome [6]. Viral genome sequencing ensures that mutations affecting pathogenicity and virulence, or those that may confer resistance to antiviral therapy are not present in the CA.

Different viral strains can be used and, based on the level of pre-existing immunity in subjects, be expected to result in differing pathogenic profiles. For viruses where the key correlates of protection are known, tests can be conducted to assess pre-existing immunity ensuring the virus produces a suitable pathogenic profile and importantly, evaluable endpoints. Sero-suitability of subjects may include testing of serum IgG, mucosal secretory IgA and T cell immunity [7–9] depending on the challenge agent of interest.

A challenge virus must be clinically relevant and present a similar pathogenic profile to that observed in a natural infection or, where appropriate, attenuated pathogenicity or disease exacerbation. For instance, rhinovirus types, A and C have been associated with asthma exacerbations, while Rhinovirus type B frequently induce a subtly different cytokine pathway leading to moderate asthma attack [10]. Wild-type viruses should always be used unless there are known risks of a severe clinical outcome. We choose viruses not deliberately attenuated but that produce symptoms consistent with clinically observed Acute Respiratory Infection (ARI) in a healthy population [11–13].

Manufacturing substrates can introduce mutations and the number of passages of the virus during manufacture should always be minimised. However, sequencing of isolates obtained during active infection in the model has shown that such mutations are rapidly selected out and thus may be of limited consequence [14].

## Pre-production and upstream processing

Viral seed material can be obtained from several sources, including an existing good manufacturing practice (GMP)-produced virus inoculum, clinical isolates or generated via reverse genetics. Careful consideration should be taken as to the source of the material regarding documentation, e.g., isolate history.

Isolates can be obtained either from subjects who acquire an infection naturally (field isolates) or from subjects inoculated experimentally (experimental challenge isolates) where the donor shows a suitable pathogenic profile. All clinical isolates must be acquired from a reputable source. The donor should be in good health with a full medical history, free from cancer or chronic illness, HIV, Hepatitis B, Hepatitis C and HTLV; nor have received blood products to mitigate the risk of any unknown pathogens and transmissible spongiform encephalopathies (TSEs).

Field isolates would likely be sourced if an appropriate seed could not be obtained from a reputable provider of virus such as the World Health Organisation (WHO), Center for Disease Control (CDC) or European Centre for Disease Control (ECDC). An isolate from an experimentally infected volunteer has advantages, the health status and medical history are known in a great level of detail as well as when in the course of infection the isolate was obtained.

The use of reverse genetics to manufacture a virus removes the need for adventitious agent testing of the donor. The appropriate pathogenicity factors can be included or eliminated from these Genetically Modified Microorganisms (GMMs). When reverse genetics is used to produce a wild-type-like virus the pathogenicity profile should be like that of the naturally occurring strain. The GMM can be deliberately attenuated; NIBRG-14 is an attenuated version of a highly pathogenic influenza (bird flu) strain with the internal genes of A/PR/8/34 (a high-yield strain) and the hemagglutinin (HA) and neuraminidase (NA) genes of A/Vietnam/1194/04(H5N1) virus. The polybasic cleavage site of the HA, known to be related to high pathogenicity, is removed [15]. Such GMMs would not be expected to provide the full pathogenic profile of a wild-type clinical strain but could still provide useful information on the efficacy of a vaccine or therapy.

Viral seed material may require pre-production processing; such upstream processing would typically involve plaque purification, rate limiting dilution, filtration and possibly selective microbial treatment.

Whenever possible, a pilot stock would be produced and screened for a range of adventitious agents as determined by the risk assessment, and as a minimum sequenced to confirm identity.

## Production and manufacturing

The manufacturing substrate will vary depending on the virus and range from embryonated eggs, cell culture and other in vivo methodologies. Each method poses challenges and risks to be assessed. Some challenge agents, including viruses and bacteria, can be propagated in Specific Pathogen Free (SPF) embryonated eggs with the advantages of low cost, ease of production and pathogen filter effect [16].

Most challenge viruses are propagated in cell culture. GMP cell lines must be fully qualified, their history and providence known; examples include Vero cells, MRC-5 cells and PerC6 cells [16–19]. Other cell lines can be used, but to reduce the risk of contamination by TSEs and latent pathogens they require extensive testing and traceability of all reagents used.

Manufacturing should be conducted in a GMP setting designed to minimise the risks involved in any production that cannot be brought to light through testing of the final product. As cited in the EU regulations [20], we consider that challenge agents should be produced on a campaign basis, which results in isolation of the CA manufacturing away from other products.

A GMP manufacturing facility should be audited both from a scientific and Quality Assurance perspective. The scientific audit determines knowledge of viral handling best practices and the Quality Assurance audit ensures appropriate regulatory requirements are met.

Any materials to be released formally from the facility must be accompanied by a copy of the complete batch record, a certificate of GMP compliance and a certificate of analysis.

## Downstream processing and testing

There are different approaches to the downstream processing of a post-GMP production harvest, but all have the same final requirements. The virus must be as free as possible from all components used during the production that may affect its final aim, i.e. a suitable pathogenicity profile while ensuring that the safety of the volunteers inoculated are not put at risk. Any product produced in a mammalian cell line should have minimal residual host cell DNA content [17, 21].

Adventitious agent testing forms a critical and substantial part of the safety data required for a challenge virus stock. The stock must be tested for any adventitious agents that could reasonably be expected to contaminate it and potentially cause a safety risk to study subjects. We do not propose a definitive specific list of adventitious agents that one should test for as the appropriate testing strategy used will depend on a range of factors as well as the current opinion of the regulator. We simply describe the best practice principles that we applied in the manufacturing of our challenge viruses and give some examples in the hope of giving some guidance to others (see Additional file 1: Tables S1 and S2).

A diluent is required to dilute the challenge stock to an appropriate inoculum titre, ensuring stability in storage before use (see Additional file 1: Figure S1 for an example of a stabilised influenza challenge virus). It must be inert, manufactured under GMP conditions and shown to be sterile, free from mycoplasma, endotoxins and other adventitious agents.

The harvest should be diluted and filled into single-use vials under GMP conditions. We propose a minimum testing panel for the release of the product from the manufacturing facility before the full adventitious testing panel required before human use (see Additional file 1: Table S1). All packaging should be suitable for clinical use, inert to the product and tamper proof. All vials should be individually labelled to enable full traceability.

Risk assessment and literature reviews to consider potential adventitious agents that could contaminate the product during each stage of the process from seed virus collection, pre-production processing, production, through to final fill of the GMP manufactured product should be conducted. For our challenge viruses, we considered the potential risks from all of the raw materials and cell banks/eggs used and possible newly emerging agents at the time of manufacture. Testing included; sterility, microbial enumeration testing, Mycoplasma & Mycobacterium detection tests along with testing for non-specific viral contaminants by in vitro inoculation of indicator cells. Also, we conducted tests for the quantification of reverse transcriptase activity as well as extensive agent-specific molecular testing for a panel of contaminants including known human viruses of both chronic and acute infections as well as the list of other potential pathogen contaminants that were highlighted by the risk assessment.

Consideration should be given to the use of an animal model to check pathogenicity. However, for ethical considerations, it should only be used when it is sure to have good correlation with human disease and provide clear guidance for subsequent human studies.

We have characterised numerous new challenge viruses following manufacture via the HVC; these included respiratory syncytial virus [13], human rhinovirus-16 [12] and influenza A/Perth/9/2009 [H3N2] [11] determining their safety, pathogenic profile and the appropriate evaluable endpoints [12, 13, 22]. These challenge viruses have been used to test the efficacy of antiviral drugs or vaccines [9, 23–29] and also the virus-host interaction [8, 13, 30–37], each time gaining more data on the challenge virus performance. It is crucial to build an extensive database of evaluable endpoints, beyond the initial characterisation studies, so as to accurately predict future performance of the virus and power subsequent product-efficacy studies.

**Fig. 1:**
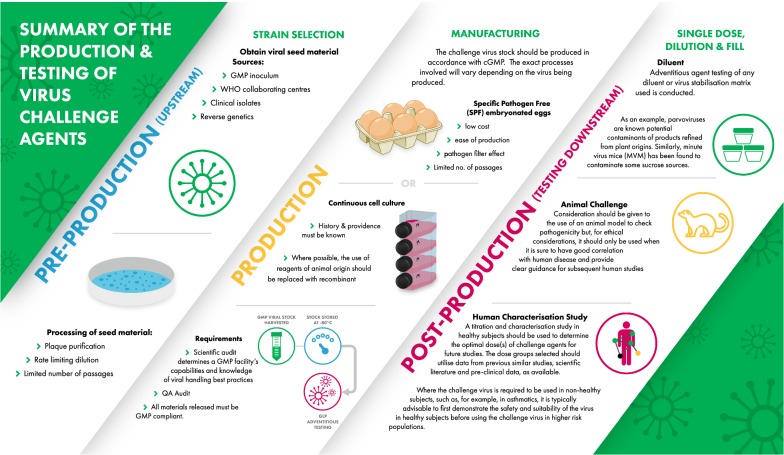
Summary of the production and testing of virus challenge agents

The production and testing of an influenza GMP challenge virus are shown schematically in Fig. 1.

## Conclusion

There are numerous decisions to be made in the complex and specialized process of manufacturing a new challenge virus with each virus production creating a unique set of circumstances to consider. This is made harder by the lack of specific regulatory and technical guidance. Therefore, we used our own direct experience in producing viral challenge agents to present an overview of the considerations and the best practice principles involved in virus manufacture so that this may serve as an initial guide to others embarking on challenge agent production. We propose that applying the methodical risk-based approach discussed above will enable new viral challenge agents to be produced, like our own, in a scientifically robust and safe manner that gives reliable results and generates clear evaluable endpoints in the HVC model. This will in turn further expand the utility of the HVC model in evaluating the efficacy of antiviral compounds and vaccines or human host interactions.

## Limitations of the manuscript

The authors acknowledge that, due to the lack of documented regulatory guidance specific to challenge virus production, the considerations and manufacturing options discussed are mainly based on the direct experience of the authors.

## Additional file

**Additional file 1: Table S1.** Minimum GMP facility Release Testing Panel, testing for egg/cell manufacturing substrates and diluent. **Table S2.** Examples of Adventitious Agent Testing panels for different scenarios. A. testing panel for 25% sucrose in PBS challenge virus diluent. B. testing panel for Allantoic fluid of control eggs. **Figure S1.** Stability of hVIVO’s A/Perth/16/2009 [H3N2] challenge virus. Each point on the graph represents a stability testing timepoint with the mean titre illustrated by the line, demonstrating ongoing infectious virus stability from the point of manufacture to latest available stability testing time point at the time of manuscript submission.

## Abbreviations

ARI: Acute Respiratory Infection
CAs: Challenge agents
CDC: Center for Disease Control
ECDC: European Centre for Disease Control
GMP: Good manufacturing practice
GMMs: Genetically modified microorganisms
HA: Hemagglutinin
NA: Neuraminidase
HVC: Human viral challenge model
FDA: US Food and Drug Administration
IND: Investigational New Drug
SPF: Specific Pathogen Free
TSEs: Transmissible spongiform encephalopathies
WHO: World Health Organisation
