# Supplementary material for: The manufacturing of human viral challenge agents for use in clinical studies to accelerate the drug development process
Source: BMC Res Notes. 2018 Aug 29;11:620. doi: 10.1186/s13104-018-3636-7 (PMC6114718; doi:10.1186/s13104-018-3636-7)
Supplement: Supplementary file 1 — Additional file 1: Table S1. Minimum GMP facility Release Testing Panel, testing for egg/cell manufacturing substrates and diluent. Table S2. Examples of Adventitious Agent Testing panels for different scenarios. A. testing panel for 25% sucrose in PBS challenge virus diluent. B. testing panel for Allantoic fluid of control eggs. Figure S1. Stability of hVIVO’s A/Perth/16/2009 [H3N2] challenge virus. Each point on the graph represents a stability testing timepoint with the mean titre illustrated by the line, demonstrating ongoing infectious virus stability from the point of manufacture to latest available stability testing time point at the time of manuscript submission. [file 13104_2018_3636_MOESM1_ESM.docx]

Table S1 Minimum GMP facility Release Testing Panel, testing for egg/cell manufacturing substrates and diluent.

| **Test** |
| --- |
| Bacteriostasis and Fungistasis |
| Sterility |
| Mycoplasma |
| Endotoxin |
| Conductivity |
| Osmolality |
| Appearance |
| pH |

Table S2: Examples of Adventitious Agent Testing panels for different scenarios. A. testing panel for 25% sucrose in PBS challenge virus diluent. B. testing panel for Allantoic fluid of control eggs

A.

| **Test** |
| --- |
| Qualification for Sterility Testing by Direct Inoculation Method (EP, USP and JP) |
| Sterility Testing by Direct Inoculation Method (EP, USP, JP) |
| Mycoplasma and Spiroplasma |
| Bovine Parvovirus type 2 |
| Bovine Parvovirus type 3 |
| Porcine Parvovirus |
| Murine Minute virus |
| LAL test for the Endotoxin (Quantitative, Kinetic, Chromogenic Assay) |
| *In Vitro* Assay for the Detection of Viral Contaminants in Viral Seed Lots and Viral Harvests in Accordance with E.P Guidelines |

.

**B.**

| **Test** |
| --- |
| Qualification for Sterility Testing by Direct Inoculation Method (EP, USP and JP) |
| Sterility Testing by Direct Inoculation Method (EP, USP, JP) |
| Mycoplasma and Spiroplasma |
| Bovine Parvovirus type 2 |
| Bovine Parvovirus type 3 |
| Porcine Parvovirus |
| Murine Minute virus |
| LAL test for the Endotoxin (Quantitative, Kinetic, Chromogenic Assay) |
| *In Vitro* Assay for the Detection of Viral Contaminants in Viral Seed Lots and Viral Harvests in Accordance with E.P Guidelines |
| Microbiological Examination of Non-Sterile Products: Microbial Enumeration Test using a spread plate method- Enumeration only |
| Microbiological Examination of Non-Sterile Products: Qualification of Microbial Enumeration Tests Using a Spread Plate Method |
| Microbial Limit with Direct Inoculation, GLP, MLT, w/o preparatory test (stasis). Testing for specific organisms (*P. aeruginosa* and *S. aureus*) per EP2.6.13 and USP 62. |
| Test for the Detection of Mycoplasmas in Accordance with the European Pharmacopoeia Section 2.6.7 |
| HA Assay for Control Eggs (EP 2.6.16) |
| In vitro Assay for the detection of ALV (Subgroups A, B and J) using CEF cells. |
| Quantification of Reverse Transcriptase Activity By Ultracentrifugation and Quantitative Fluorescent Product Enhanced Reverse Transcriptase (QFPERT) Assay*. |
| Avian Leukosis virus A |
| Avian Leukosis virus B |
| Avian Leukosis virus C |
| Avian Leukosis virus D |
| Avian Leukosis virus E |
| In Vitro Assay for the Detection of Viral Contaminants in Viral Seed Lots and Viral Harvests in Accordance with E.P Guidelines |

*ALV E is an endogenous retrovirus of birds and is, therefore, commonly detected in eggs both as a positive result in the specific ALV E test and the assay for Reverse Transcriptase detection. However, ALV E is non-infectious to humans so positive detection is acceptable for egg-produced challenge viruses.

Additional Figure S1

**Additional Figure 1**: Stability of hVIVO’s A/Perth/16/2009 [H3N2] challenge virus. Each point on the graph represents a stability testing timepoint with the mean titre illustrated by the line, demonstrating ongoing infectious virus stability from the point of manufacture to latest available stability testing time point at the time of manuscript submission.
